# One size does not fit all: the influence of age at surgery on outcomes following Norwood operation

**DOI:** 10.1186/1749-8090-9-100

**Published:** 2014-06-14

**Authors:** Tara Karamlou, Kristen Sexson, Andrea Parrish, Karl F Welke, D Michael McMullan, Lester Permut, Gordon Cohen

**Affiliations:** 1 Division of Pediatric Cardiac Surgery, Benioff Children’s Hospital, University of California, San Francisco, 513 Parnassus Avenue, Suite S-549, California, CA 94143, USA; 2 Division of Pediatric Cardiology, Texas Children’s Hospital, Houston, TX, USA; 3 Division of Pediatric Cardiac Surgery, Children’s Hospital of Illinois, Peoria, Il, USA; 4 Section of Pediatric Cardiothoracic Surgery, University of Washington School of Medicine and Seattle Children’s Hospital, Seattle, WA, USA

**Keywords:** Norwood, Single ventricle, Outcomes, Risk-factors, Congenital heart disease

## Abstract

**Background:**

Given our large catchment area that often results in later presentation age, we sought to understand our institutional outcomes for the Norwood operation in the context of published data. Specifically, we studied whether operative and late death post-Norwood are dependent on age at operation.

**Methods:**

Retrospective review of 105 consecutive infants undergoing Norwood (2004–2011) at our institution. Patients were divided into those undergoing Norwood ≤ 7 days of age (N = 43; 41%) and those undergoing Norwood > 7 days of age (N = 63; 59%). Operative mortality (≥30 days), interstage mortality (between Norwood and superior bidirectional Glenn), STS-mortality (operative death + in-hospital death), and late mortality, occurring any time following hospital discharge were compared among groups. Multivariable factors for mortality at each time-point were compared using logistic regression models.

**Results:**

Underlying diagnosis was HLHS in 67 (64%) with the remainder (N = 38; 36%) being other single ventricle variants. Median age at surgery was 8 days (range 1–63 days) and mean weight at surgery was 3.2 ± 0.6 kg. Pulmonary blood flow was provided by a right ventricle-pulmonary artery conduit in 94% (N = 99). Overall operative survival was 92%, with 73% (N = 66) undergoing bidirectional Glenn. Median age was higher for operative survivors compared to non-survivors (12 days vs. 5 days; *P* = 0.036), with operative mortality higher for infants ≤7 days at Norwood compared to infants >7 days at Norwood (14% vs. 3%; *P* = 0.04). After censoring for in-hospital death, age ≤ 7 days was also associated with increased risk for late death (26% vs. 5%; *P* = 0.005).

**Conclusions:**

In contrast to other institutional series, infants at our center undergoing Norwood operation at an earlier age have worse outcomes. Adoption of published practice patterns could lead to different local outcomes because of intangible center-specific effects, underscoring the principle that results from one institution may not be generalizable to others. Targeted center-specific internal review, if possible, should precede externally recommended changes in practice.

## Background

Investigation of the influence of surgical timing on early outcomes following operative repair for complex congenital heart disease in term neonates has not led to consensus [1-7]. Several single-center studies have found that younger age at surgery leads to improved outcomes following Norwood operation [1-7]. However, other studies have reported that younger age at surgery leads results in suboptimal physiologic parameters and higher mortality [2,4]. Given our large catchment area that often results in later patient age at admission, we sought to understand our institutional outcomes in context of published data and determine whether operative and late death post-Norwood were dependent on age at operation.

## Methods

Approval was obtained from the institutional review board at Seattle Children’s Hospital. A retrospective review of our institution’s Society of Thoracic Surgeons’ database identified 105 consecutive infants undergoing Norwood operation between 2004 – 2011 at Seattle Children’s Hospital. Additional data pertinent to the study were abstracted from individual patient charts. Patients were included with all types of anatomy that eventuated in Norwood reconstruction as initial palliation regardless of their planned repair (univentricular versus biventricular) algorithm. Echocardiograms reviewed by a single echocardiologist (KS) to document morphology. Patients were divided into the following age groups: patients ≤ 7 days of age at surgery and those > 7 days of age at surgery. Because a main impetus of the current study was to test the hypothesis of generalizability using age at Norwood operation as a platform, this age cut-point was chosen to allow comparison to recently published single-institution data. Mortality was compared between groups at the following time-points: 1) operative mortality (defined as death occurring at ≤ 30 days); 2) interstage (between Norwood and bidirectional Glenn); 3) any state mortality following discharge from hospital. In addition, because the Society of Thoracic Surgeons (STS) has recently, and appropriately for inclusive reasons, changed their preferred definition of operative mortality to: “1) all deaths, regardless of cause, occurring during the hospitalization in which the operation was performed, even if after 30 days (including patients transferred to other acute care facilities); and 2) all deaths, regardless of cause, occurring after hospital discharge, but before the end of the 30^th^ postoperative day”, we tabulated mortality in accordance with this definition. Multivariable logistic regression analysis determined risk-factors for mortality at these three cross-sectional time-points. Significance was considered to be *P* < 0.05. SAS software, version 9.3 (Cary, NC) was used for all analyses.

## Results

### Patients

We identified 105 patients from our institution that met the eligibility criteria over the study period. Underlying diagnosis was hypoplastic left heart syndrome (HLHS) in 67 (64%) with the remainder (N = 38; 36%) being other single ventricle variants. Median age at surgery was 8 days (range 1–63 days) and mean weight at surgery was 3.2 ± 0.6 kg. Pulmonary blood flow was provided by a right ventricle-pulmonary artery conduit in 94% (N = 99). Forty three (41%) of patients were ≤ 7 days and 62 (59%) were > 7 days at the time of Norwood reconstruction. Median age for those ≤ 7 days was 5 days (range 1–7), and 12 days (range 8–63) for those > 7 days of age. Patient characteristics differed slightly among groups, Table 1. Importantly, preoperative creatinine was higher and there were a greater number of patients with hypoplastic left heart syndrome (HLHS) among those patients ≤ 7 days of age compared to the older neonates. There was a trend toward a higher prevalence of noncardiac anomalies and a higher preoperative hematocrit among the younger neonates compared to those > 7 days of age, though statistical significance was not reached. The median age of patients was lower (5 days) for patients who died at ≤ 30 days compared to those who survived (12 days).

**Table 1:** Patient characteristics

**Variable**	**≤ 7 days N = 43 (41%)**	**> 7 days N = 62 (59%)**	**P-value**
Female	44%	43%	0.87
Weight at operation (kg)	3.2 ± 0.5	3.2 ± 0.6	0.97
Preoperative creatinine (mg/dl)	0.6 ± 0.1	0.5 ± 0.1	0.02
Preoperative hematocrit (%)	42 ± 5	40 ± 6	0.05
H LHS	77%	55%	0.02
Non-cardiac anomaly	16%	5%	0.05
AVV moderate or greater	9.5%	22%	0.26
Prenatal diagnosis	81%	59%	0.01
Restrictive atrial septum	32%	21%	0.25
Pulmonary venous obstruction	0%	4%	0.31

### Patient outcomes

**Figure 1:**
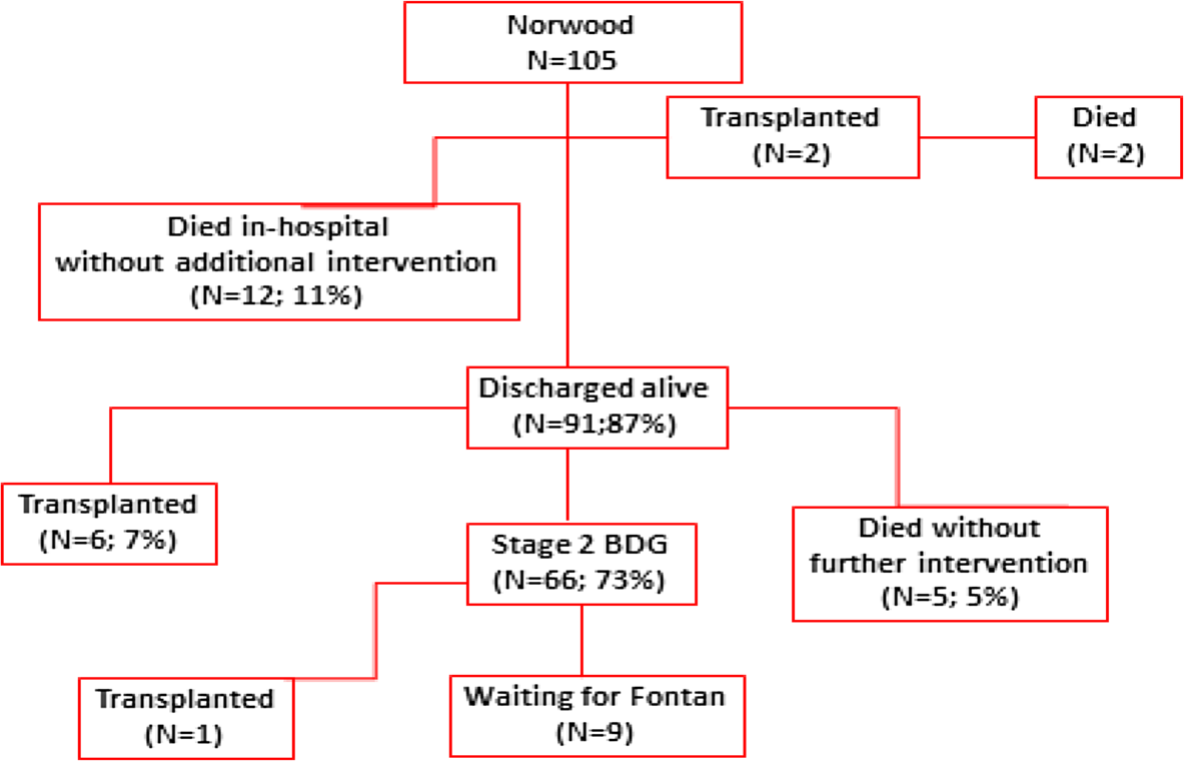
**Flow-chart of events for 105 infants undergoing Norwood operation.** BDG, bidirectional Glenn.

**Figure 2:**
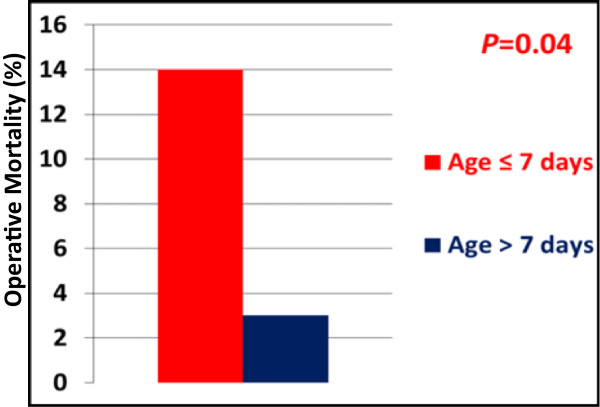
**Histogram showing operative mortality (≤30 days) for patient groups.** Infants undergoing Norwood operation (red bar) ≤ 7 days had significantly higher mortality compared to those > 7 days of age at Norwood (blue bar).

**Figure 3:**
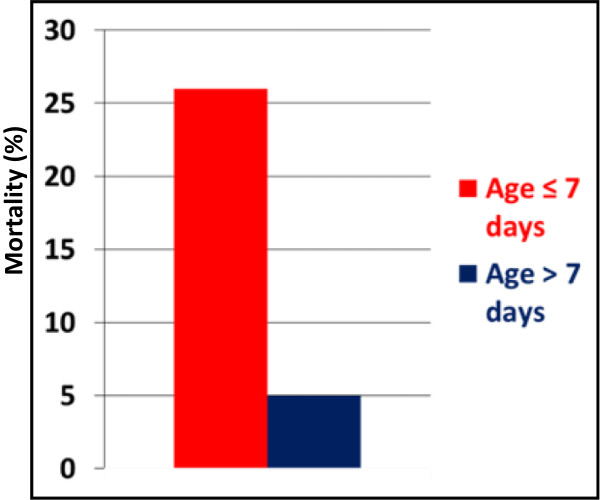
**Histogram showing mortality at any time following hospital discharge for patient groups.** Infants undergoing Norwood operation ≤ 7 days (red bar) had significantly higher mortality compared to those > 7 days of age at Norwood (blue bar).

A flow chart of patient events following Norwood is shown in Figure 1. Overall there were 26 deaths (25%). Twenty-one deaths were among patients operated on within the first 3 years, with only 5 deaths since 2008. Three patients died (2 in-hospital and 1 late) following transplantation. Operative mortality was 8% (n = 8) and was significantly lower for those patients undergoing Norwood at > 7 days of age compared to the younger neonates, Figure 2. In-hospital mortality was 11% (n = 12). Using the STS definition for operative mortality, there were 14 (13%) deaths. There was a trend toward lower STS-defined mortality among the older patients [10% (6/62) for patients > 7 days of age vs. 19% (8/43) for patients ≤ 7 days of age; *P* = 0.18}, though statistical significance was not reached. Similarly, overall any-state death post discharge was also significantly lower for patients > 7 days of age (5%) vs. those ≤ 7 days (26%), *P* = 0.005, Figure 3. While not statistically significant, interstage death (16% overall) also tended to be lower for patients > 7 days of age (11%) vs. those ≤ 7 days (23%), *P* = 0.10.

### Multivariable risk-factors

We were not able to identify any multivariable factors for death at any of the time-points examined. Importantly, neither a diagnosis of HLHS, year of surgery, the presence of noncardiac anomalies (both of which were more prevalent in the younger cohort), nor younger age at surgery were associated with mortality at any time-point.

## Discussion

Our study, using age at surgery as a platform, illustrates the concept that recommendations based on results at one institution may not be generalizable to another institution. We have shown that infants undergoing Norwood operation at age > 7 days of age have improved early and intermediate outcomes compared to those infants undergoing surgery at age ≤ 7 days. While we are not advocating delaying operative intervention beyond 12 days (which was the median age among our older subgroup), the present data *do* suggest that variation in practice patterns (so-called intangible factors including differences in ICU care or operative management) may preclude direct translation of treatment guidelines between centers. An earlier report from our institution demonstrated that univentricular palliation in infants could be performed in infants > 30 days of age with acceptable results (zero in-hospital mortality) [8]. Importantly, two of the 9 patients in this small case series died following bidirectional Glenn. We acknowledge that late outcomes are incompletely studied in the present study due to the immature state of the cohort. While our operative mortality (9.7%) and interstage mortality (16%) rates are comparable to other contemporary results [9-12], including those from the recently published Single Ventricle Reconstruction Trial (11.5% for 30-day mortality and 12% for interstage mortality, respectively), there could be higher late attrition related to age at initial Norwood. Our results are in contrast to previously published studies, many of which showed older age to be an important risk-factor for post-Norwood mortality [3-7]. Alsoufi et al. [4] reported outcomes from 55 infants older than 14 days at their center. They found that age < 3 weeks at Norwood was a risk factor for both mortality prior to bidirectional Glenn and for overall mortality, but that those infants > 2 weeks required pulmonary vasodilators and higher inotropic support that translated into ongoing attrition following bidirectional Glenn [4]. Similarly, Hehir et al. [3] studied 313 hospital survivors following Norwood operation at the Children’s Hospital of Philadelphia. They showed that age at operation > 7 days was a risk-factor for interstage death. Importantly, the presence of intact atrial septum, which was more prevalent in the older infants, was also strongly associated with interstage death. An older report from Mahle and colleagues [5], also from the Children’s Hospital of Philadelphia, found that age > 14 days at surgery was associated with higher mortality following Norwood.

Determining a physiologic or rational explanation for our results is challenging in the context of a retrospective study in which treatment patterns ostensibly changed during the course of the study. Inadequate statistical power precluded a fruitful multivariable analysis that included both era as well as the other anatomic and ypsiologic parameters. Results at our institution undoubtedly improved over the study period despite year of surgery being an insignificant univariable predictor of outcomes. Regarding potential physiologic influences, Tweddell and colleagues reported that neonates undergoing Norwood operation earlier than 8 days of age had lower superior vena cava saturations (as a surrogate for mixed venous saturations) and wider arterial-venous oxygen difference in the first 48 hours following Norwood reconstruction. It is possible that older neonates had improved systemic-pulmonary flow matching due to a less dynamic pulmonary vascular bed. It is also possible that younger patients had a trend toward a higher prevalence of noncardiac anomalies as well as a higher prevalence of an underlying diagnosis of HLHS, which, in other studies have led to worse outcomes following Norwood reconstruction [9,12].

### Limitations

The results reported are from a retrospective review of a prospectively maintained institutional dataset. Furthermore, due to a paucity of events, we were unable to identify multivariable factors associated with outcome. Our selected groups differed with respect to specific factors (including an underlying diagnosis of HLHS) that could have biased results in favor of the older cohort. However, these specific factors also were not identified to be informative with respect to outcome in our univariable analysis. Similarly, though year of surgery was not significant in univariable analysis, results clearly improved over time indicating that era may have been an important factor. Our dichotomization of the variable age, though apparently arbitrary, was pragmatic, insofar as it was chosen to allow comparison to other previous published studies while allowing enough patients in each group. Finally, later outcomes, which arguably could be adversely impacted by later age, were incompletely studied in the present report.

## Conclusion

In contrast to other institutional series, infants at our center undergoing Norwood operation at an earlier age have worse outcomes. Adoption of published practice patterns could lead to different local outcomes because of intangible center-specific effects, underscoring the principle that results from one institution may not be generalizable to others. Targeted center-specific internal review, if possible, should precede externally recommended changes in practice.

## Abbreviations

STS: Society of Thoracic Surgeons; HLHS: Hypoplastic left heart syndrome.

## Competing interests

The authors declare that they have no competing interests.

## Authors’ contributions

All authors shared in the conception and execution of the study. TK completed the data analysis, primarily authored the manuscript, and was responsible for completing the revisions and submitting the manuscript. KW and AP assisted with the statistical analysis and critically reviewed the manuscript and made editorial changes. AP was also involved in abstracting and interpreting the data sets. KS performed all of the echocardiographic reviews and entered all of the offline echocardiographic information into the data spreadsheet. DM, LP and GC were responsible for idea conception, manuscript preparation, editing, and for final interpretation of the data. All authors read and approved the final manuscript.
